# Recommencement of atezolizumab with associated pulmonary sarcoid‐like reaction

**DOI:** 10.1002/rcr2.1363

**Published:** 2024-04-26

**Authors:** Khai Tam, Michael Wallace, Siobhain Mulrennan

**Affiliations:** ^1^ Respiratory and Hepatology Department Sir Charles Gairdner Hospital Perth Western Australia Australia

**Keywords:** atezolizumab, immune checkpoint inhibitor, recommencement, sarcoid‐like reaction, sarcoidosis

## Abstract

An 81 year old male with Child‐Pugh A cirrhosis and metastatic hepatocellular carcinoma (HCC) treated with 3‐weekly atezolizumab and bevacizumab developed a pulmonary sarcoid‐like reaction (SLR) after 5 months. Atezolizumab, an immune checkpoint inhibitor, was identified as the likely culprit. He was treated with prednisolone, resulting in improvement, and was successfully rechallenged with both atezolizumab and bevacizumab.

## Graphical abstract

An 81 year old male with Child‐Pugh A cirrhosis and metastatic hepatocellular carcinoma (HCC) treated with 3‐weekly atezolizumab and bevacizumab developed a pulmonary sarcoid‐like reaction (SLR) after 5 months. Atezolizumab was identified as the likely culprit. He was treated with prednisolone, resulting in improvement, and was successfully rechallenged with both atezolizumab and bevacizumab.

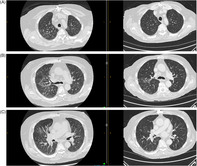

## INTRODUCTION

An 81 year old male with a history of Child‐Pugh A liver cirrhosis secondary to alcohol complicated by metastatic hepatocellular carcinoma(HCC) in 2020, was treated with 3‐weekly atezolizumab and bevacizumab. After 5 months, he developed sarcoid‐like pulmonary reaction, with atezolizumab(immune checkpoint inhibitor) identified as the culprit. He was treated with prednisolone, resulting in improvement, and was successfully rechallenged with both atezolizumab and bevacizumab.

There has been a few case studies regarding sarcoidosis‐like reactions(SLR) induced by immune checkpoint inhibitors.^1^ These reactions are indistinguishable from sarcoidosis, exhibiting similar clinical manifestations and histology. They are commonly observed in the skin and intrathoracic areas. The median time between the initiation of immune checkpoint inhibitors and development of SLR averaged 14 weeks. These reactions usually resolve with withdrawal of immunotherapy or with the initiation of steroid therapy. Currently, there is no concrete understanding of the pathophysiology underlying SLR triggered by checkpoint inhibitors. Limited data exist on the successful re‐introduction of immune checkpoint inhibitors in pulmonary SLR.

## CASE REPORT

**FIGURE 1:**
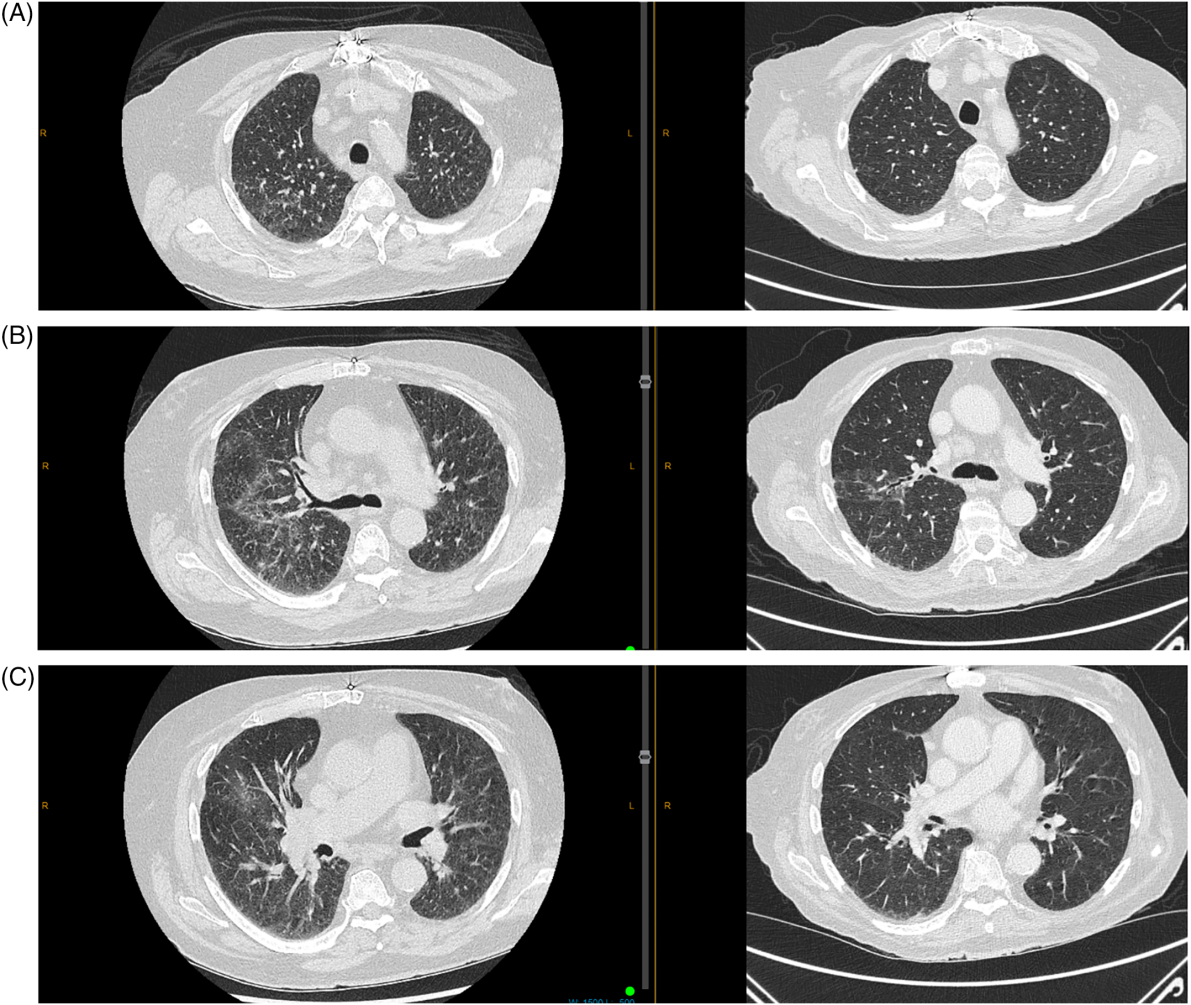
(A) Initial HRCT May 2021 (Left), Follow up HRCT October 2021 (right) Upper zone. (B) Initial HRCT May 2021 (Left), Follow up HRCT October 2021 (right) Mid zone. (C) Initial HRCT May 2021 (Left), Follow up HRCT October 2021 (right) Lower zone.

**FIGURE 2:**
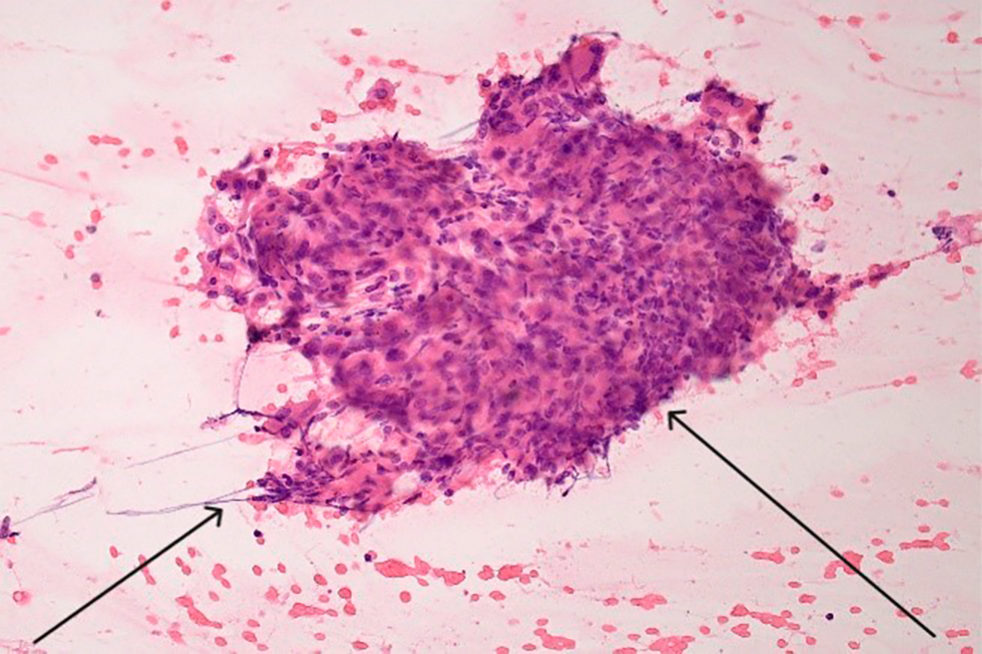
Non‐necrotising granulomas composed of aggregates of epithelioid histiocytes and admixed multinucleate giant cells.

An 81‐year‐old gentleman with a history of Child‐Pugh A liver cirrhosis secondary to alcohol, complicated by metastatic hepatocellular carcinoma (HCC) in 2020, also had a background of type 2 diabetes, retinal haemorrhage, aortic valve replacement, hypercholesterolemia, hypertension, and a 10‐pack‐year history of smoking in his 20s. His medication history included metoprolol, metformin, rosuvastatin, dapagliflozin and lercanidipine. He was commenced on atezolizumab and bevacizumab in December 2020 for his metastatic HCC in his porta hepatis lymph node. After 5 months of immunotherapy, during which he exhibited a partial response, a CT scan in May 2021 was done to investigate the development of a dry cough associated with weight loss of 6 kg. The CT changes were suggestive of sarcoidosis, and consisted of intrathoracic lymphadenopathy and widespread bilateral micronodules in a perilymphatic and peribronchovascular distribution (Figure 1A–C). He had no infective symptoms. His serum calcium was normal at 2.38 mmol/L. Bibasal crepitations were detected on chest examination, with normal oxygen saturation levels on room air and respiratory rate. Atezolizumab and bevacizumab infusions were withheld, and he subsequently underwent an endobronchial ultrasound‐guided transbronchial needle aspiration of his lymph nodes in June 2021. The procedure revealed non‐necrotizing, well‐formed granulomas (Figure 2) with no evidence of lymphoma or Acid fast bacilli detection, confirming the diagnosis of SLR likely caused by atezolizumab. Following this, his pulmonary function tests revealed a mild restrictive ventilatory defect with a total lung capacity (TLC) of 72% predicted, forced expiratory volume 1 (FEV1) of 77% predicted, forced vital capacity (FVC) of 71% predicted and an FEV1/FVC ratio of 81%. His diffusing capacity for carbon monoxide (DLCO) was 72% pf predicted and carbon monoxide transfer coefficient (KCO) of 107% predicted. He was started on prednisolone in June 2021, with 50 mg/day for 2 weeks, weaning to 25 mg/day for the next 2 weeks. A repeat CT chest in a month showed significant improvement in his pulmonary infiltrates and his cough had also resolved. He was subsequently weaned off prednisolone over a period of 3 weeks and restarted on atezolizumab and bevacizumab in August 2021 during the prednisolone weaning period. His follow‐up CT chest in October 2022 (Figure 1A–C) revealed almost complete resolution of his interstitial infiltrates. He continued with 3‐weekly atezolizumab and bevacizumab until January 2023, with minimal adverse effects, when it was ceased due to the complete response of his metastatic disease. He is currently undergoing interval MRI scans for monitoring.

## DISCUSSION

Atezolizumab is an anti‐PD‐L1 monoclonal antibody immune checkpoint inhibitor with an established role in oncological therapy. It is indicated for the treatment of metastatic hepatocellular carcinoma, metastatic melanoma, and lung cancers. Immune checkpoint inhibitor‐associated SLR are clinically and histologically identical to sarcoidosis, characterized by focal infiltration of noncaseating epithelioid granulomas. Atezolizumab has been associated with SLR in a phase 1 trial, with one patient out of 207 developing SLR.^1^ In cases of pulmonary SLR, symptoms may include fatigue, fever, dyspnea, and cough. While SLR can be multisystemic, it predominantly involves intrathoracic organs and the skin.^2^ The onset of SLR can vary from 3 weeks to 2 years,^3^ and there is no clear dose threshold for the development of checkpoint inhibitor‐related SLR.^3^ The pathophysiology of this phenomenon is currently uncertain, but the association is drawn from the linear causality upon initiation of checkpoint inhibitors. Tissue biopsy is necessary to distinguish SLR from other causes of immune‐related adverse events (irAEs), such as pneumonitis or reactive lymphadenopathy. Biopsy is also important to avoid misinterpretation of the CT abnormalities as progression of metastatic disease and treatment failure, which could be dire. SLR is known to be steroid‐responsive and often resolves upon discontinuation of immune checkpoint inhibitors.^4^ Corticosteroid treatment may not be required in certain cases of SLR but is usually started for symptomatic patients or if it is prudent to continue with immunotherapy despite SLR due to its beneficial effect.^3^

Regarding the recommencement of immune checkpoint inhibitors following the resolution of SLR, evidence is limited, especially in the context of atezolizumab and pulmonary SLR. One case report demonstrated no progression in SLR after 8 months.^5^ It has been suggested that SLR from checkpoint inhibitors may indicate an effective antitumor response, potentially serving as a predictive parameter of treatment response.^5^ This possibility seems plausible in our patient, who subsequently demonstrated a remarkable treatment response to immunotherapy. Therefore, acknowledging the possibility of reinstituting immune checkpoint inhibitors, especially when the SLR is mild, is important.

In conclusion, recognizing the association of pulmonary SLR with immune checkpoint inhibitors is crucial. We appreciate that immune checkpoint inhibitor‐mediated pulmonary SLR can be managed with steroids and the withdrawal of the causative agent. We acknowledge the potential for reinstituting checkpoint inhibitors following the resolution of SLR, especially when the patient responds well to immunotherapy. We also postulate that SLR may be a predictive sign of the efficacy of checkpoint inhibitors but further studies are required.

## AUTHOR CONTRIBUTIONS

Khai Tam (main author), Siobhain Mulrennan (Supervisor), and Michael Wallace (Supervisor).

## CONFLICT OF INTEREST STATEMENT

None declared.

## ETHICS STATEMENT

The authors declare that appropriate written informed consent was obtained for the publication of this manuscript and accompanying images.

## Data Availability

The data that support the findings of this study are available from the corresponding author upon reasonable request.
